# Silver nanoparticles induced testicular damage targeting *NQO1* and *APE1* dysregulation, apoptosis via *Bax/Bcl-2* pathway, fibrosis via *TGF-β*/*α-SMA* upregulation in rats

**DOI:** 10.1007/s11356-022-23876-y

**Published:** 2022-11-11

**Authors:** Doaa H. Assar, Abd-Allah A. Mokhbatly, Mohamed F. Abou ELazab, Emad W. Ghazy, Ahmed A. Gaber, Zizy I. Elbialy, Ayman A. Hassan, Ahmed Nabil, Samah Abou Asa

**Affiliations:** 1 Clinical Pathology Department, Faculty of Veterinary Medicine, Kafrelsheikh University, Kafrelsheikh, 33516 Egypt; 2 Department of Fish Processing and Biotechnology, Faculty of Aquatic and Fisheries Sciences, Kafrelsheikh University, Kafrelsheikh, 33516 Egypt; 3 High Technological Institute of Applied Health Sciences, Egypt Liver Research Institute and Hospital (ELRIAH), Sherbin, ElMansora Egypt; 4 Beni-Suef University, Beni-Suef, Egypt, Egypt Liver Research Institute and Hospital (ELRIAH), Sherbin, ElMansora Egypt; 5 Pathology Department, Faculty of Veterinary Medicine, Kafrelsheikh University, Kafrelsheikh, 33516 Egypt

**Keywords:** Silver nanoparticles, Testes, Sperm parameters, Oxidative stress, Apoptotic pathway

## Abstract

In medicine, silver nanoparticles (AgNPs) are employed often. They do, however, have negative impacts, particularly on the reproductive organs. This research aimed to assess AgNP impact on the testis and the possible intracellular mechanisms to induce testicular deteriorations in rats at various concentrations and different time intervals. Sprague Dawley rats (*n* = 40) were allocated into four equal groups: the control one, and three other groups injected intra-peritoneally with AgNP solution 0.25, 0.5, and 1 mg/kg b.w. respectively for 15 and 30 days. Our findings revealed that AgNPs reduced body and testicular weights, estradiol (E2) and testosterone (T) hormone levels, and sperm parameters while elevating the nitric oxide and malondialdehyde levels with inhibition of reduced glutathione contents in testicular tissue. Interestingly, AgNPs significantly upregulated the testicular inducible nitric oxide synthase, B cell lymphoma 2 (Bcl-2)-associated X, transforming growth factor, and alpha-smooth muscle actin (α-SMA) expression levels. However, apurinic/apyrimidinic endo deoxyribonuclease 1 (APE1), NAD (P) H quinone dehydrogenase 1 (NQO1), and Bcl-2 expression levels were all downregulated indicating exhaustion of body antioxidant and repairing defense mechanisms in testicles in comparison with the control rats. Various histological alterations were also detected which dramatically increased in rats sacrificed after 30 days such as loss of the lining cells of seminiferous tubules with no spermatozoa and tubular irregularities associated with thickening of their basement membranes. Immunolabeling implicated in the apoptotic pathway revealed a negative expression of Bcl-2 and marked immunoreactivity for caspase-3 after 30 days of AgNP treatment in comparison to the control rats. To our knowledge, there have been no previous publications on the role of the α-SMA, APE1, and NQO1 genes in the molecular pathogenesis of AgNP testicular cytotoxicity following AgNP acute and chronic exposure.

**Graphical Abstract:**

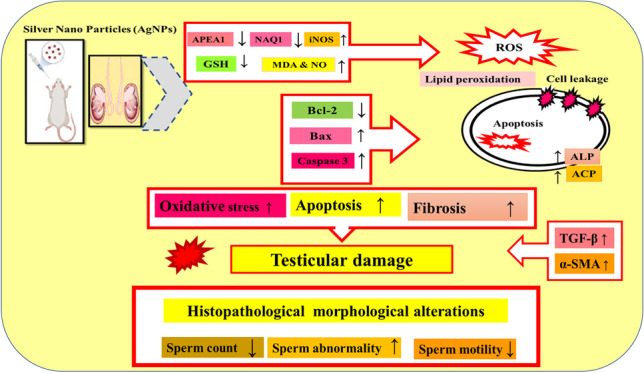

**Supplementary Information:**

The online version contains supplementary material available at 10.1007/s11356-022-23876-y.

## Introduction

Currently, the usage of nanoparticles (NPs) and nano-delivery systems in medicine is expected to spread quickly and become more relevant in the medical industry for medication delivery and diagnosis applications (Habas et al. 2021). Silver nanoparticles are extensively utilized in consumer products such as cell phones, toothbrushes, medical tools, scalpels, several medical products, and wound dressings (Yoshida et al. 2018). However, regardless of several advantages of NPS, their potential health hazards cannot be neglected due to their uncontrollable use, emission to the natural environment, and potential toxic effects (Khan et al. 2019), and possible contribution as a threat to human health (Massarsky et al. 2014). External toxicants and even NPs have been found to make the male reproductive organ sensitive to environmental stress (Tang et al. 2019). When nanoparticles are directly exposed in vitro or injected in vivo, they have diverse effects on sperm cell activities (Shittu et al. 2018). The ability of nanoparticles to pass the hemato-testicular barrier has been proven, raising questions concerning their systemic dispersion and biocompatibility (Wang et al. 2018). Nanoparticles induce the reduction of reactive oxygen species (ROS) that cause oxidative stress (OS) when there is imbalance in the redox state of the cell (Sakr et al. 2017). One of the most important reasons for male infertility is oxidative stress (Hussein et al. 2016) and is thought to be the reason for nanotoxicity (Ema et al. 2017). Silver nanoparticle treatment has cytotoxic effects on both Leydig and Sertoli cells in a way hindering spermatogenesis. The effects of AgNPs on the various parameters of rat sperm are size- and dose-dependent (Miresmaeili et al. 2013). Moreover, Garcia et al. (2014) showed that AgNPs significantly alter steroid production and impact testis health. Asare et al. (2012 and 2016) found that AgNPs caused genotoxicity and DNA damage in the testicular cells. A very few studies have evaluated the toxic effects of sublethal doses of NPs for a short period of exposure (Lamberti et al. 2014). Recent studies demonstrated that most NPs have adverse actions on male germ cells (Braydich-Stolle et al. 2010; Ahmed et al. 2017). However, the effects of AgNPs on testes morphology, sex hormone levels, and sperm production and underline mechanism are still unclear. The present study aimed to assess the potential effects of AgNPs at different doses (0.25, 0.5, and 1.0 mg/kg b.w.) for a short period (15 successive days) and long period (30 successive days) on the hormonal assay, semen parameters, testicular oxidative stress, antioxidant status, and histological changes in addition to immunohistochemical. Although the molecular mechanisms underlying AgNP administration at different doses are unknown, we also performed gene expression analysis for some genes targeting oxidative stress and apoptosis.

## Materials and methods

### Statement of ethics

The experiment was approved by the Faculty of Veterinary Medicine, Kafrelsheikh University, and Egypt’s Institutional Animal Care and Animal Ethics Committee. During the experiment, every precaution was taken to minimize animal pain.

### Synthesis of silver nanoparticles

The following procedure was used to make silver nanoparticles: An oil bath was used to heat 25 mL of 6.8 mM tri-sodium citrate in an aqueous solution containing 7 mM tannic acid to 60 °C. After heating, the solution was added to 100 mL of 0.74 mM AgNO_3_ that had previously been pre-heated to 60 °C with vigorous stirring. The solution was held at 60 °C for a few minutes until the color changed to yellow. The combination was then held at 97 °C for another 45 min before cooling to room temperature and being stored at 4 °C in the dark (Bastús et al. 2014).

#### Silver nanoparticle characterization

**Fig. 1:**
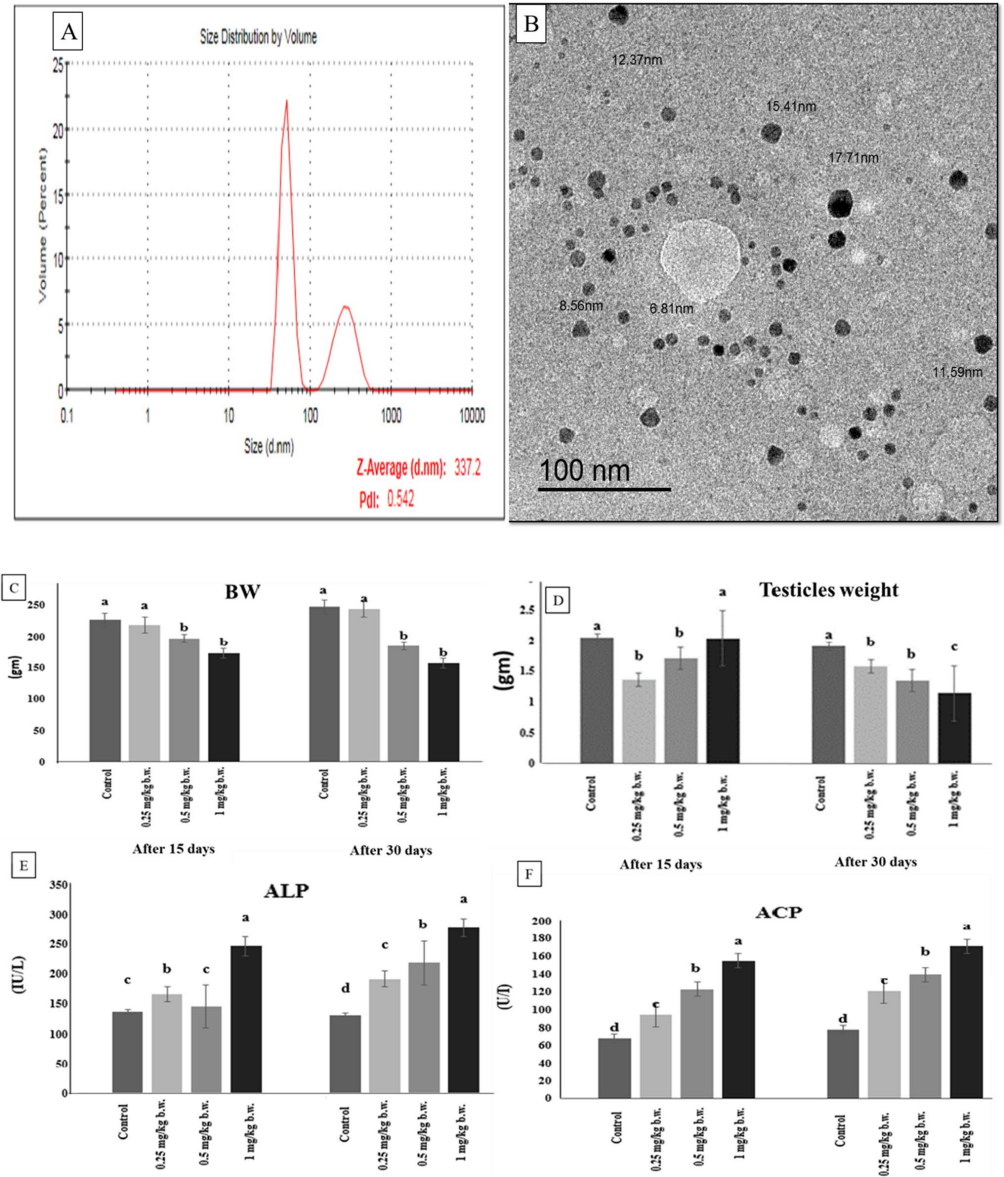
(**A**) Characterization of silver nanoparticles (AgNPs) using Malvern ZETA Sizer Nano series: it shows particles average size 337.2 with poly dispersion index 0.542. (**B**) Characterization of silver nanoparticles (AgNPs) using transmission electron microscopy images: it shows fine dispersion of spherical like shape particles in the samples with size ranged from (6.81 to 17.71 nm) at 100 nm scale. (**C**) Body weight (BW), (**D**) testicular weight changes, (**E**) alkaline phosphatase (ALP), and (**F**) acid phosphatase (ACP) enzymatic activities of the control and AgNP-treated groups after 15 and 30 days. Data are expressed as mean ± SEM (*n* = 7). Subscript letters (a, b, c, and d) indicate that means with different superscripts are significantly different at *p* < 0.05

The functional features of the produced particles must be evaluated; hence, AgNPs must be characterized. We used two different analytical approaches to characterize the generated AgNPs: dynamic light scattering (DLS) and transmission electron microscopy (TEM) as shown in (Fig. 1).

##### Dynamic light scattering

It is a strategy that relies on light’s interaction with particles. It can be used to measure narrow particle size distributions, particularly in the 2–500-nm range. In aqueous or physiological fluids, it is primarily employed to determine particle size and size distributions (Fig. 1(A)). The size acquired by DLS is frequently bigger than that obtained by TEM, which could be attributable to Brownian motion (Zhang et al. 2016). At the Electron Microscopy Unit at Mansoura University in Egypt, we use the Malvern ZETA Sizer nano series.

##### Transmission electron microscopy

At the Electron Microscopy Unit, Mansoura University, Egypt, samples were put on carbon-coated Cu grids (200 mesh) and studied using a JEM 2100 electron microscope (JEOL, Tokyo) at 200 kV using HRTEM and an ORIUS Gating camera (Fig. 1(B)).

### Experimental animals

Forty male Sprague Dawley rats (10 weeks old, weighing 150–200 g) were obtained from Mansoura University’s Medical Experimental Research Center. They were kept in well-ventilated animal rooms in stainless steel cages with sterilized rice husk as bedding. Before beginning the experiment, they were acclimated for 1 week in a controlled environment (temperature 24 2 °C; humidity 60%; light 12-h light–dark cycle) with free access to water and a regular pellet diet ad libitum. The experimental procedure was approved by the Kafrelsheikh University Faculty of Veterinary Medicine in accordance with the rules established by the Institutional Animal Ethical Clearance committee.

### Experimental design

After a 1-week acclimation period, the experimental animals were placed into four groups (ten rats for each):

1. The control group was given 0.9% normal saline intravenously.
2. Group with a low AgNP dose (0.25 mg AgNPs/kg b.w.).
3. Group with a medium AgNP dose (0.5 mg AgNPs/kg b.w.).
4. Group with high AgNP dose group (1.0 mg AgNPs/kg b.w.) according to Qin et al. (2017) and Mirzaei et al. (2017).

Each animal group was divided into two subgroups, each containing five rats. The first subgroup received a daily dose of AgNPs for 15 days and the second subgroup received an AgNP dose daily for 30 days. The experimental plan is depicted in Figure S1.

### Sampling

Animals were weighed and euthanized with 75 mg/kg b.w. thiopental sodium (EMEA 1999) through cardiac puncture at each specified exposure period (15 and 30 days). Blood samples were taken via the retro-orbital venous plexus in plain tubes, clotted, then exposed to centrifuge at 3000 rpm at 4 °C for 15 min, followed by serum separation. Clear serum samples were stored in Eppendorf tubes at − 80 °C until it was utilized to measure serum alkaline phosphatase (ALP), acid phosphatase (ACP), estradiol, and testosterone following the manufacturer’s protocol. Testicles were removed, cleaned, and washed with cold saline for each rat. The testicles were dissected and weighed separately. A portion of each rat’s left testis was homogenized in cold phosphate buffer saline (PBS). The homogenates were centrifuged for 10 min at 4 °C at 3000 rpm. The obtained supernatants were kept at − 20 °C. The second specimen from the left testis was fixed in solution of 10% formalin solution for histological examination, while the other part was frozen at − 80 °C for oxidative stress biomarkers, antioxidant status, and gene expression evaluation.

### Semen evaluation

On a wormed, clean glass slide, cauda epididymis was sliced into small pieces and incised to liberate spermatozoa. Two microliters of released spermatozoa was combined with 20 μL of 2.9% sodium citrate and coverslipped (Fathi et al. 2019). At least two microscopic areas were examined at 400 × magnification to determine the percentage of motile sperm. A hemocytometer was used to estimate the sperm count, which was seen at a magnification of 40 × . The eosin-nigrosine stain was used to evaluate the proportion of viable and aberrant spermatozoa in seminal smears.

### Biochemical assay

ACP and ALP activities, estradiol, and testosterone have been all detected in serum.

### Testicular oxidative and antioxidant status

The frozen testicle tissues (about 1 g) were thawed, washed in ice-cold KCl solution (1.15%), weighed, and then homogenized separately in ice-cold 4 volumes of homogenizing buffer (1.15% KCl with 50 nM Tris–HCl to obtain pH at 7.4). Each sample was separately homogenized, and the contents were centrifuged at 10,000 × *g* for 20 min in a centrifuge (Sigma 2–16 k). The supernatant layer was then separated, and preserved at − 20 °C. After, the reaction with thiobarbituric acid lipid peroxide was measured and expressed as nanomole (nmol) malondialdehyde (MDA) per tissue weight. Nitric oxide (NO) level in the testicular homogenate was determined as total nitrite/nitrate. Reduced glutathione (GSH) concentration in testicular tissue was also determined. All kits were purchased from Biodiagnostics, Cairo, Egypt.

### Histopathology

Tissue sections from each rat’s testes were processed for histopathological examination. The sections were fixed directly in 10% formalin, dehydrated in alcohols, cleared in xylene, and embedded in paraffin blocks. Hematoxylin and eosin–stained sections of 5-m thickness were obtained. For the identification of collagen fibers, 5-m-thick paraffin sections were prepared and stained with Masson trichrome.

### Bcl-2 and Caspase-3 immunohistochemical expression in testicular tissues

B cell lymphoma 2 (Bcl-2) and Caspase-3 immunohistochemical staining was performed on testicular tissue samples from rats given 1 mg AgNPs for 30 days by using 4-μm-thick paraffin-embedded sections. Deparaffinization in xylene was followed by rehydration in graded ethanol. The sections were immersed in 0.05 M citrate buffer, pH 6.8 solution for antigen retrieval. Endogenous peroxidase was inhibited for 20 min at room temperature by incubating in 0.3% H_2_O_2_ in methanol. At room temperature, protein Block Serum Free was applied to the sections for 30 min to prevent non-specific protein binding. Immunolabeling was done overnight in a humidified chamber at 4 °C with rabbit monoclonal anti-BcL-2 antibodies (Abcam, Cat# ab182858, at a dilution of 1:500) and polyclonal anti-caspase 3 antibodies (Invitrogen, Cat# PA5-77,887, at a dilution of 1:100). After washing with PBS, the sections were incubated for 30 min at room temperature with a goat anti-rabbit secondary antibody (Cat# K4003, EnVision + TM System Horseradish Peroxidase Labelled Polymer; Dako). After washing with PBS, the sections were incubated for 30 min at room temperature with a goat anti-rabbit secondary antibody (Cat# K4003, EnVision + TM System Horseradish Peroxidase Labelled Polymer; Dako). After washing in distilled water, the sections were counterstained with Mayer’s hematoxylin, dehydrated in an alcohol gradient, cleared with xylene, and mounted for examination under a light microscope. Bcl-2 and Caspase-3 immunoreactivities were graded as follows: the ratio of spermatogonia and spermatocytes with positive immunostaining was calculated by counting 1000 cells in 10 high-power fields (× 400). By counting 1000 cells in 10 high-power fields, the immunoreactivities of Bcl-2 and Caspase-3 were scored as follows: negative, 0–10% positive cells; weakly positive, 10–25% positive cells; moderately positive, 25–50% positive cells; and strongly positive, > 50% positive cells (× 400). The ratio of immunopositive spermatogonia to spermatocytes was calculated. The proportion of Bcl-2 and Caspase-3 positivity was compared using the *T*-test, and *P* < 0.05 was considered significant.

### RNA extraction and qRT-PCR

About 100 mg of testicle tissues was rinsed in sterilized phosphate-buffered saline before being homogenized in liquid nitrogen with a Teflon and pestle homogenizer and stored at − 80 °C until RNA isolation. Total RNA was isolated using Trizol (iNtRON Biotechnology) as directed by the manufacturer. The Maxime RT PreMix (Oligo dT primer) was used to create cDNA from purified RNA (iNtRON Biotechnology, Korea). The reaction mixture, which included RNA and master mix, was heated to 45 °C before being inactivated at 95 °C. SensiFast SYBR Lo-Rox kit (Bioline) Master Mix was used to perform Q-rtPCR for the target genes. Table 1 shows the primer sequences for all target and reference genes, as well as the PCR conditions (Table 1) and they are nitric oxide synthase 2 (*iNOS*), transforming growth factor (*TGF-β*), alpha-smooth muscle actin (*αSMA*), BCL-2-associated X (*BAX*) and *BCl-2*, apurinic/apyrimidinic endodeoxyribonuclease 1 (*APE1*), and NAD(P)H quinone dehydrogenase 1 (*NQO1*) genes. The fold change of mRNA expression was calculated using the 2^−ΔΔCt^ method (Livak and Schmittgen 2001) after recording the Ct values for reference and target genes.

**Table 1:** Sequences of the used primers for the real-time PCR analysis

Gene	Primer sequence 5′-3′	NCBI accession number	Reference
GAPDH	F:CAGCAATGCATCCTGCAC R:GAGTTGCTGTTGAAGTCACAGG	XM_017592435.1	Nakahara et al. (2004)
iNOS	F-CTACCTACCTGGGGAACACCTGGG R-GGAGGAGCTGATGGAGTAGTAGCGG	S71597.1	Hori et al. (2001)
αSMA	F-CGATAGAACACGGCATCATC R-CATCAGGCAGTTCGTAGCTC	NM_031004.2	Ghassemifar et al. (1997)
BAX	F-GTTGCCCTCTTCTACTTTGC R-ATGGTCACTGTCTGCCATG	NM_017059.2	Sadek et al. (2016)
BCl-2	F-CCCCAGAAGAAACTGAACC R-GCATCTCCTTGTCTACGC	NM_016993.1	Sadek et al. (2016)
APE1	F: TGCTGTGTGGGGATCTCAAT R: CCAACATTCTTAGAGCGGGC	NM_024148.1	Sadek et al. (2016)
NQO1	F:ACCTCTCTGTGGTTTAGGGC R:GGACCTGGGTGTGCTATGTA	NM_017000.3	Xie et al. (2018)

### Statistical analysis

Data are expressed as mean ± standard errors of means (SEM) and statistically analyzed using SPSS (Version 22, IBM Corp.). Prior to analyzing, data were tested for normality and homogeneity using Shapiro–Wilk’s test and the Levene’s test, respectively. Analysis of variance (ANOVA) test followed by Tukey’s multiple comparison test was run to determine the significant differences among experimental groups at probability error of 5% (*p* < 0.05).

## Results

### Characterization of the applied AgNPs

Figure 1 represents a laser diffraction particle size analyzer (Zeta sizer NanoZS90, Malvern, UK) was used to observe and image the morphology and size distribution of AgNPs in deionized water and suspension. According to the scans, AgNPs with an average size of 12.0 nm has a spherical shape.

### Body, testes weights, and biochemical assay

A statistically significant decrease (*p* ≤ 0.05) occurred in body weight differences between the control and all treatment groups, particularly rats injected with 0.5 and 1 mg/kg AgNPs in a dose-dependent relationship, with the lowest value observed in the rats’ group receiving 1 mg AgNPs on days 15 and 30 of the experiment when compared to the control group. On the other hand, rats injected with 0.25 mg AgNPs showed a non-significant change in the b.w. all over the experimental period (Fig. 1(C)). There is a significant (*p* ≤ 0.05) decrease in the testis’ weight in the low-dose group and medium-dose group after 15 days, but more pronounced in the low-dose group while the high-dose group did not show any significant changes compared with the control group. On day 30 of the experiment, there was a significant (*p* ≤ 0.05) decrease in testicle weight in all groups compared to the control group, and it was clear that there is a dose relationship, as the lowest values were recorded at the end of the experiment in the high dose group (Fig. 1(D)). Furthermore, serum ALP and ACP enzyme activities were significantly increased (*p* ≤ 0.05) in AgNP-treated groups than in the control group following 15 and 30 days. The highest activities were observed in the AgNPs high-dose group when compared to the control group (Fig. 1(E, F)). Data concerning the b.w., testicle weights, ALP, and ACP are seen in Fig. 3.

### Sperm parameters

**Fig. 2:**
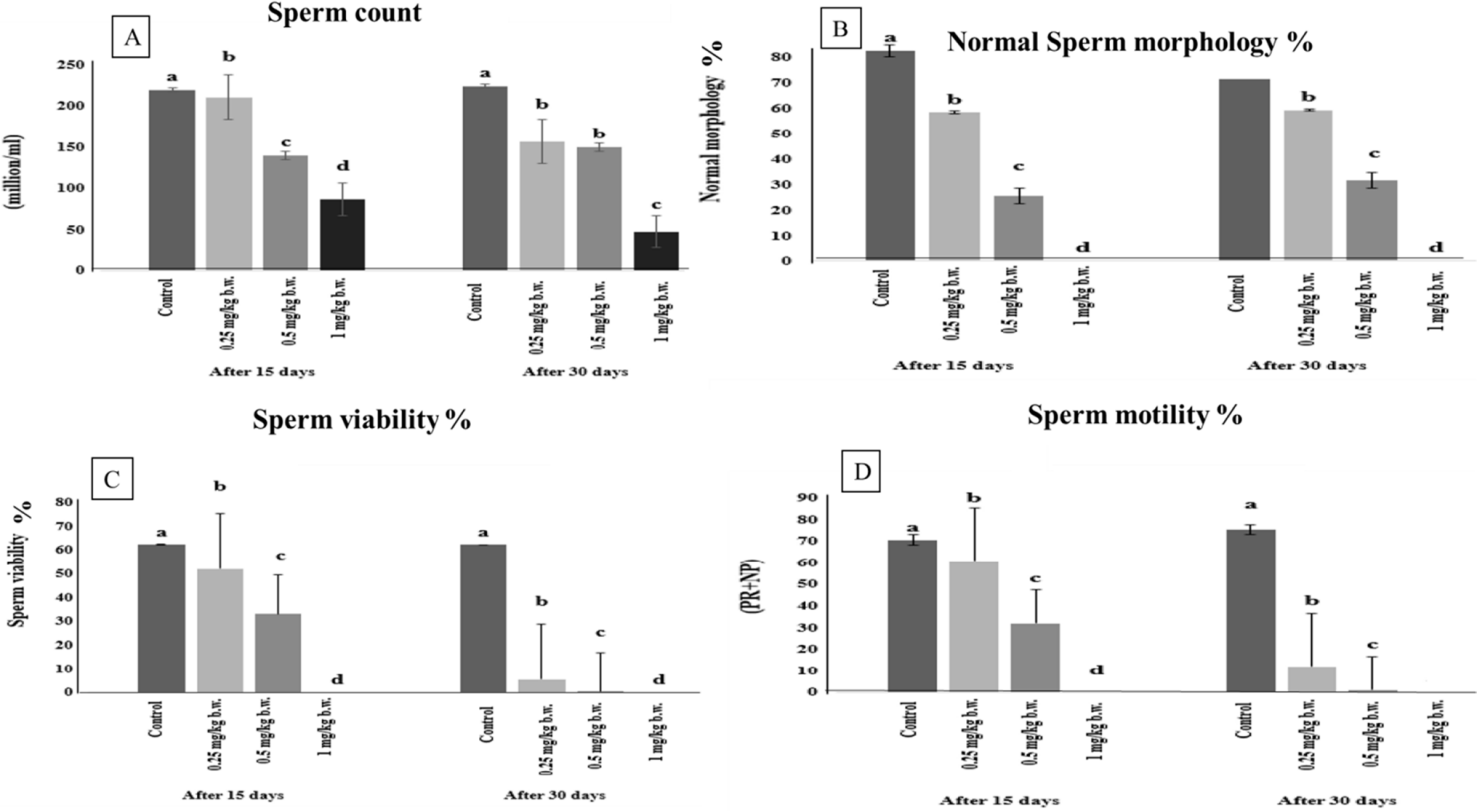
Semen picture in control and AgNP-treated groups after 15 and 30 days of i.p administration. (**A**) Sperm count, (**B**) sperm morphology, (**C**) sperm viability, and (**D**) sperm motility. Data are expressed as mean ± SEM (*n* = 7). Superscript letters (a, b, c, and d) indicate that means with different superscripts are significantly different at *p* < 0.05

AgNP-treated groups showed a significant reduction (*p* ≤ 0.05) in the sperm count, normal morphology, viability, and motility in all AgNP-treated groups after days 15 and 30 of the experiment compared with the control group with a dose relationship, as the high AgNP group (1 mg/kg) showed the most significant decrease in the abovementioned parameters than the other treated groups compared with the control group at the days 15 and 30 of the experiment. Figure 2 portrayed the semen picture in the control and AgNP-treated groups.

### Hormonal assay and oxidative stress and antioxidant biomarkers evaluation

**Fig. 3:**
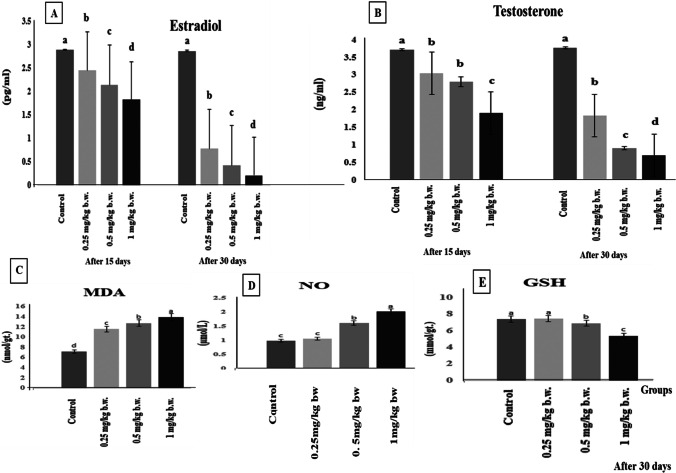
(**A**) Serum estradiol hormone, (**B**) testosterone hormone after 15 and 30 days of AgNP injection. (**C**) Testicular oxidative biomarkers malondialdehyde (MDA), (**D**) nitric oxide (NO), and (**E**) reduced glutathione (GSH) of the control and AgNP-treated groups after 30 days of AgNP injection. Data are expressed as mean ± SEM (*n* = 7). Superscript letters (a, b, c, and d) indicate that means with different superscripts are significantly different at *p* < 0.05

Furthermore, AgNP administration revealed a significant (*P* ≤ 0.05) decline in estradiol (E2) and testosterone (T) hormones levels when compared to the control group post 15 and 30 days, as the rats group given 1 mg/kg AgNPs recorded the lowest significant levels than the other treated groups compared to the control group on days 15 and 30 of the experiment (Fig. 3(A, B)). Moreover, the testicular level of MDA was significantly elevated (*p* ≤ 0.05) following AgNP administration in all treated groups after 30 days with a dose relationship, as the rat group administered with 1 mg/kg AgNPs recorded the highest significant values (*p* ≤ 0.01) as compared with the control group among the other treated groups (Fig. 3(C)). The NO values showed a significant (*p* ≤ 0.05) increase in medium- and high-dose groups after 30 days compared with the control group, but the high-dose group was more significant (*p* ≤ 0.01) than the medium-dose group, and the low-dose group showed a slight increase in NO values than the control group but not significant (Fig. 3(D)). When compared to the control group, the testicular content of GSH was significantly lower in AgNP-treated groups. The reduction in GSH level was more obvious in the rats that received 1 mg AgNPs for 30 days in comparison to the control group, while the low-dose group did not show any significant changes all over the experimental period compared to the control group (Fig. 3(E)). Changes in MDA, NO, and GSH are illustrated in Fig. 5(C).

### Histopathology

#### Testicular histopathological changes after 15 days of AgNP exposure

**Fig. 4:**
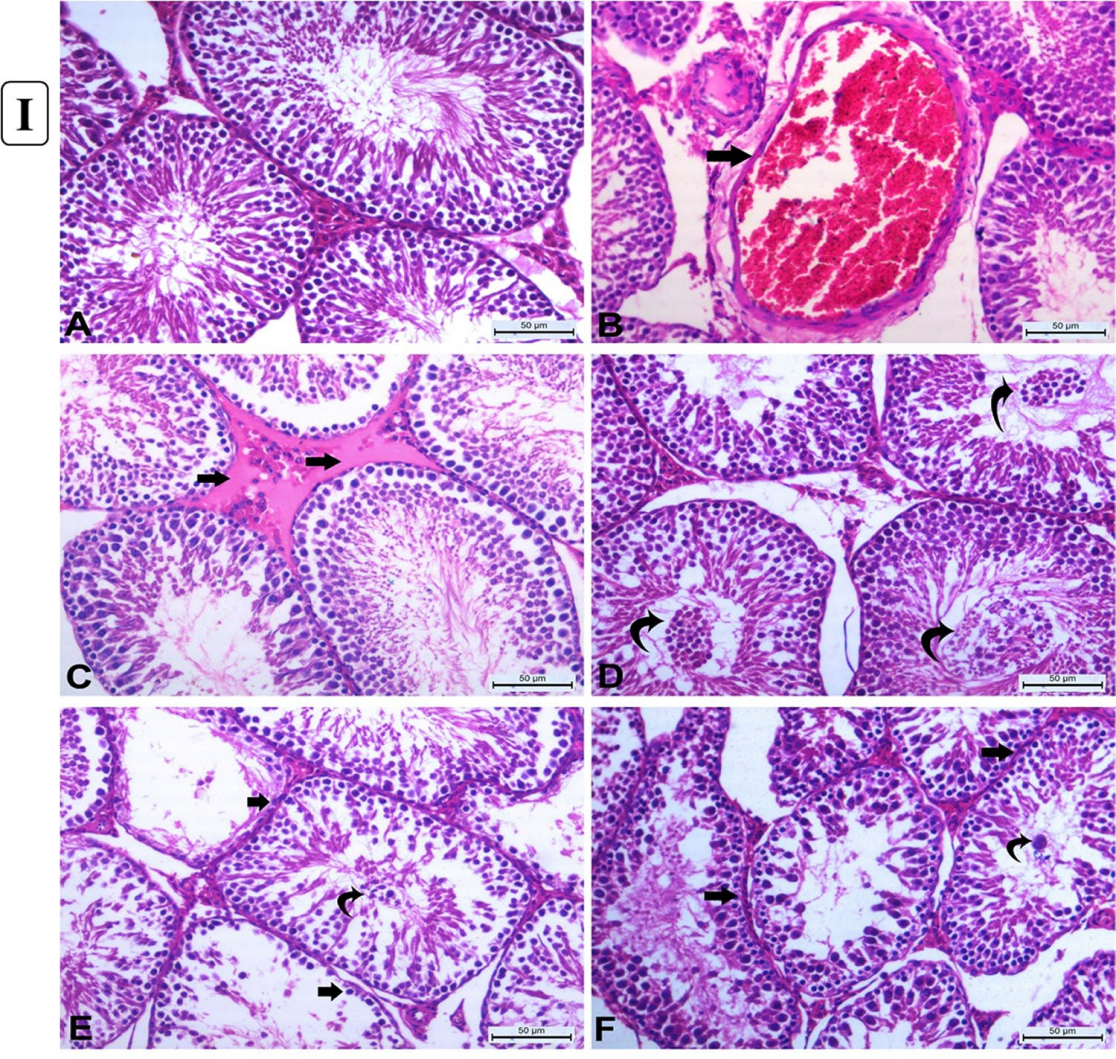

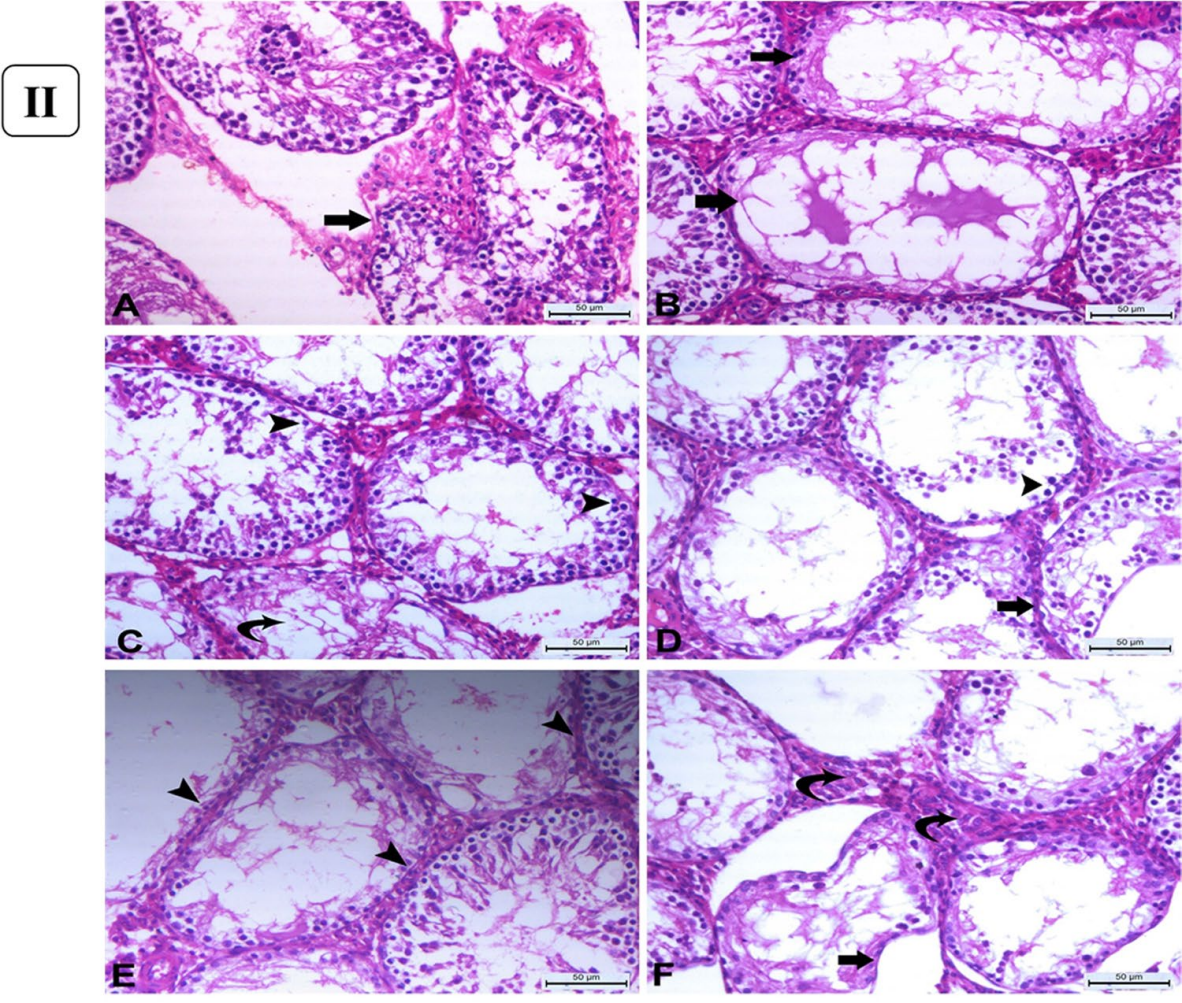
(I) Histopathology of rats’ testicular tissues after 15 days of AgNP exposure. (**A**) Control rats showing normal seminiferous tubules with normal orderly arranged spermatogenic cells and active spermatogenesis with normal clusters of Leydig cells. (**B**) Rats received 0.25 mg/kg b.w. AgNPs showing marked congestion of interstitial blood vessel (arrow). (**C**) Rats received 0.25 mg/kg b.w. AgNPs showing interstitial edema as a faint eosinophilic material (arrows). (**D**) Rats received 0.5 mg/kg b.w. AgNPs showing increasing degenerative changes in the germinal lining of seminiferous tubules and shedding into the lumen (*curved arrows*). (**E**) Rats received 1 mg/kg b.w. AgNPs showing increased degeneration of the lining of seminiferous tubules which have with few to no spermatogonia cells (*arrows*) and sloughing of the germinal epithelium into the lumen of seminiferous tubules (*curved arrow*). (**F**) Rats received 1 mg/kg b.w. AgNPs showing irregularities of the contour of the degenerated seminiferous tubules with thickened basement membranes (arrows) with the presence of spermatid giant cell( curved arrow) increasing the number of seminiferous tubules with degenerative changes in the germ cells. *All are H&E stained (*× *200), bar* = *50µ*. (II) Histopathology of rats’ testicular tissues after 30 days of AgNP exposure. (**A**) Rats received 0.25 mg/kg b.w. AgNPs showing increasing the irregularities of seminiferous tubules (arrow) with vacuolar degeneration of spermatogonia and primary spermatocytes with sloughed degenerated spermatogenic cells into the lumen with impaired spermatogenesis. (**B**) Rats received 0.25 mg/kg b.w. AgNPs, some seminiferous tubules showing obvious degenerative alterations, with only pyknotic spermatogonial cells being the only lining cells (arrows). (**C**) Rats received 0.5 mg/kg b.w. AgNPs showing increased number of seminiferous tubules showed marked degeneration of their lining with pyknosis of their nuclei (*arrow head*) with complete hyalinization of some tubules (*curved arrow*). (**D**) Rats received 0.5 mg/kg b.w. AgNPs showing many seminiferous tubules lined with few pyknotic spermatogonia cells reveal substantial degenerative alterations (*arrowhead*) associated with thickening of their basement membrane (*arrow*) without active spermatogenesis. (**E**) Rats received 1 mg/kg b.w. AgNPs showing degenerated tubules devoid of germinal epithelium or with individual pyknotic spermatogonia with thickened basal lamina (*arrowheads*). (**F**) Rats received 1 mg/kg b.w. AgNPs showing marked distortion and atrophy of seminiferous tubules devoid of spermatozoa (arrow), as well as hyperplasia of Leydig cell clusters (*curved arrows*) with some tubules lined only with pyknotic spermatogonia and Sertoli cells. *All are H&E stained (*× *200), bar* = *50µ*

Control rat testis histological sections revealed normal seminiferous tubules with normal orderly arranged spermatogenic cells and active spermatogenesis with normal clusters of Leydig cells (Fig. 4(I.A)). However, rats injected with AgNPs for 15 days demonstrated dose-dependent histological changes. The most noticeable changes in rats given 0.25 mg/kg body weight were mild congestion (Fig. 4(I.B)) and interstitial edema as a faint eosinophilic material (Fig. 4(I.C)). Mild degeneration changes of germ cells which desquamated into the lumen were observed. Also, the thickening of the basement membrane of some tubules with the slight proliferation of Leydig cells was seen. The number of seminiferous tubules showed epithelial degenerative and desquamative changes were increased in rats treated with 0.5 mg/kg b.w. AgNPs (Fig. 4(I.D)) together with increasing the number of tubules devoid of spermatozoa with the widening of inter-tubular spaces. Furthermore, in rats received 1mg/kg b.w., AgNPs showed increases in the number of degenerated tubules lined with few to no spermatogenic cells (Fig. 4(I.E)) with the presence of multinucleated spermatid giant cells (Fig. 4(I.F)). Some other tubules showed vacuolar degeneration of spermatogenic cells. All histopathological changes observed in the testes of rats in the different experimental groups are summarized in Table 2.

**Table 2:** Histopathological changes in testes of rats injected intraperitoneally with different concentrations of AgNPs for 15 days post-AgNP administration

Lesions after 15 days of exposure	(0.25 mg/kg b.w.)	(0.5 mg/kg b.w.)	(1.0 mg/kg b.w.)
Congestion	+ +	+	+
Interstitial edema	+ +	+	+
Vacuolar degeneration	+	+	+ +
Tubular degeneration/desquamation	+	+ +	+ +
Tubular necrosis	+	+	+ +
Retarded spermatogenesis	*** − ***	+	+

− , negative; + , mild; +  + , moderate; +  +  + / +  +  +  + , severe

#### Testicular histopathological changes after 30 days of AgNP exposure

The rats were given 0.25 mg/kg b.w. after 30 days of AgNP administration which revealed irregularities and shrinkage of some seminiferous tubules. These tubules exhibited degenerative changes and lacked spermatozoa (Fig. 4(II.A)). Other tubules showed complete loss of their spermatogenic cells with vacuolar changes in their lumen (Fig. 4(II.B)). However, another group of seminiferous tubules showed marked degenerative changes to complete loss of their spermatogenic cells except for very few spermatogonia and Sertoli cells. By increasing dose, the rats received 0.5mg/kg b.w. AgNPs revealed increasing the frequency of irregular-shaped seminiferous tubules; these tubules were elongated with marked degeneration (Fig. 4(II.C)) where only a few pyknotic spermatogonia cells with a deeply stained nucleus and vacuolated cytoplasm are the only lining cell with thickening of their basement membrane (Fig. 4(II.D)). Some tubules showed marked vacuolar degeneration of spermatogenic cells. Also, increase in Leydig cell clusters was observed. At higher doses, the rats received 1mg/kg b.w. AgNPs; degenerative and necrotic changes became very notable in a wide number of seminiferous tubules with thickening of their basement membranes accompanied with inactive spermatogenesis (Fig. 4(II.E)) with some tubules only Sertoli cells are present. The seminiferous tubules became atrophied and devoid of all their spermatogenic cells (Fig. 4(II.F)) with marked thickening of their basement membrane and increased Leydig cell clusters. However, congestion of small inter-tubular blood vessels was observed. Also, silver brown pigments were detected. All these pathological lesions are shown in Table 3.

**Table 3:** Histopathological changes in testes of rats injected intraperitoneally with different concentrations of AgNPs for 30 days post-AgNP administration

Lesions after 30 days of exposure	(0.25 mg/kg b.w.)	(0.5 mg/kg b.w.)	(1.0 mg/kg b.w.)
Congestion	*** − ***	*** − ***	+
Interstitial edema	*** − ***	*** − ***	*** − ***
Vacuolar degeneration	+	+ +	+ +
Tubular degeneration/desquamation	+	+ +	+ + +
Tubular irregularities	+	+ +	+ + +
Tubular necrosis/atrophy	+ +	+ + +	+ + + +
Retarded spermatogenesis	+	+ +	+ + +
Brown pigment	*** − ***	*** − ***	+

− , negative; + , mild; +  + , moderate; +  +  + / +  +  +  + , severe

#### Masson’s trichrome staining

**Fig. 5:**
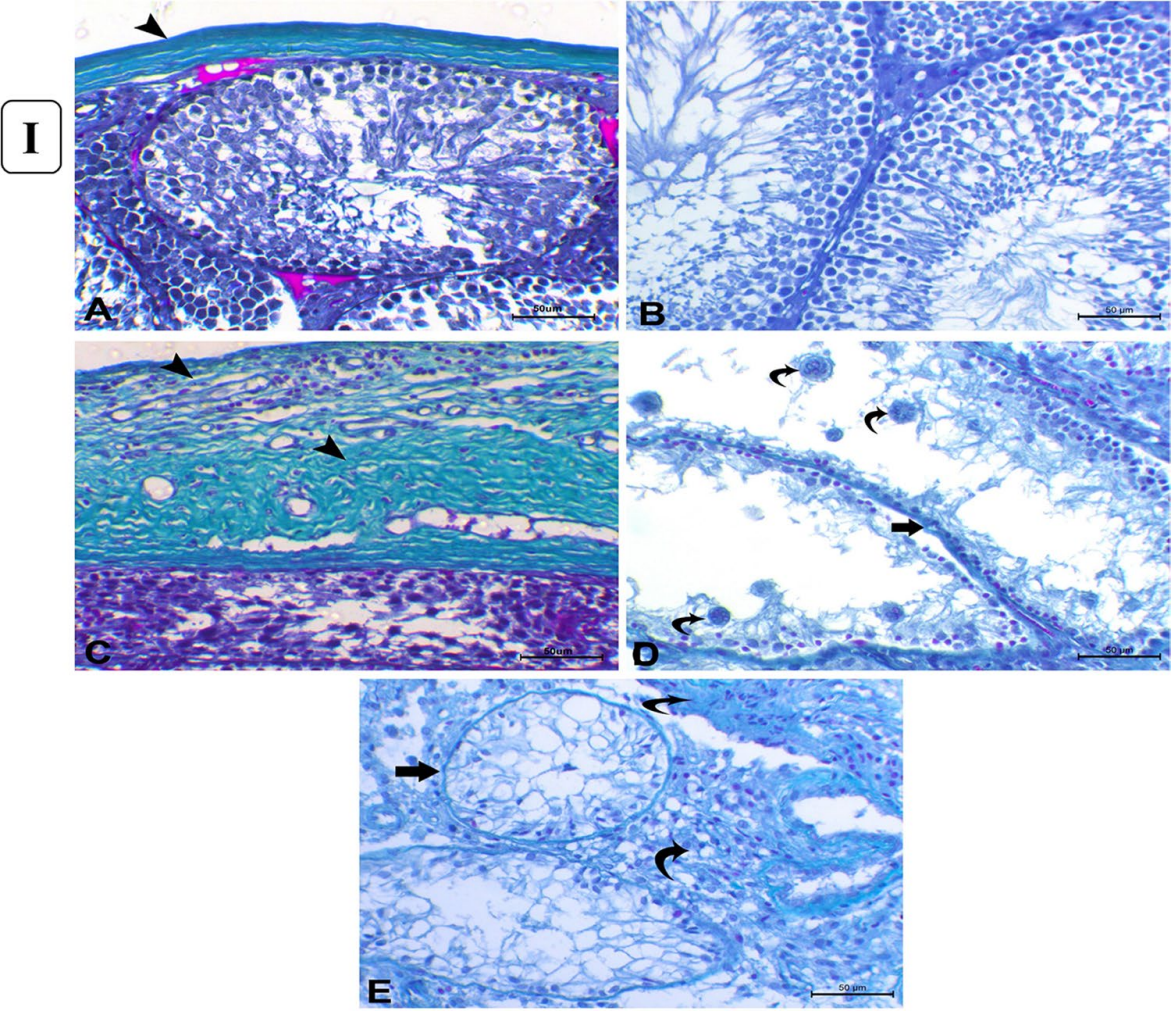

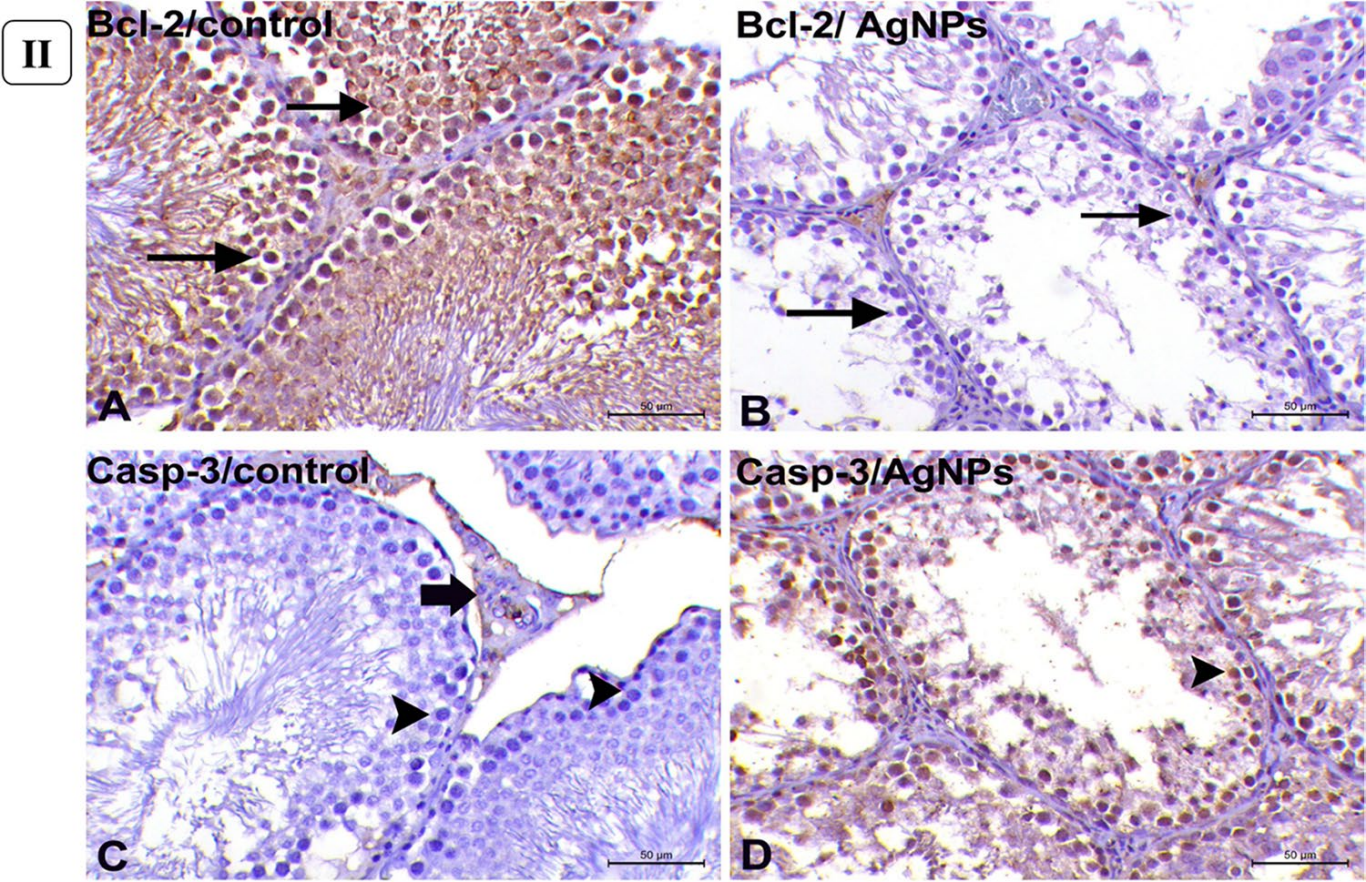
(I) Histopathology of Masson’s trichrome-stained sections of the testes. (**A**) Control group showing normal thin layer of tunica albuginea which is greenish in color (*arrowhead*). (**B**) Control group showing minimal amount of collagenous fibers in the interstitial tissue between the seminiferous tubules. (**C**) AgNP rats group injected with 1 mg for 30 days showing marked thickening of tunica albuginea (*arrowheads*). (**D**) AgNP rats group injected with 1 mg AgNPs for 30 days showing thickening of the basement membrane of the degenerated seminiferous tubules (*arrow*) with the presence of multiple spermatid giant cells (*curved arrows*). (**E**) AgNP rats group injected with 1 mg AgNPs for 30 days showing thickening of tubular basement membrane of completely degenerated and atrophied seminiferous tubules (*arrow*) with excessive amount of collagenous fibers in interstitial tissue (*curved arrows*). (*Masson’s trichrome staining* × 200), *bar* = *50µ.* (II) Immunohistochemical expression of Bcl-2 and Caspase-3 in rats’ testicular tissues after exposure to 1 mg AgNPs for 30 days. (**A**) Control testes, showing strong positive cytoplasmic expression of Bcl-2 (*arrows*). (**B**) AgNP-treated group, showing negative expression of Bcl-2 (*arrows*) except for endothelium of small blood vessels in the interstitium. (**C**) Control testes, showing negative cytoplasmic immunolabeling of Caspase-3 (*arrowheads*) except for some Leydig cells (*arrow*). (**D**) AgNP-treated group, testes showing strong cytoplasmic labeling of Caspase-3 (*arrowhead*). Immunohistochemistry (IHC × 200, bar = 50µ)

Furthermore, Masson’s trichrome-stained sections of the control testes showed a thin layer of tunica albuginea (Fig. 5(I.A)) associated with basement membrane thickening in severely degenerated seminiferous tubules (Fig. 5(I.B)). However, rats administered with 1mg/kg b.w AgNPs for 30 days showed marked thickening of tunica albuginea (Fig. 5(I.C)) associated with thickening of the basement membrane of the markedly degenerated seminiferous tubules (Fig. 5(I.D)) and increasing the number of collagenous fibers in the interstitial tissue between the atrophied seminiferous tubules (Fig. 5(I.E)).

### Bcl-2 and caspase-3 immunolabeling in testicular tissues

In the cytoplasm of the lining cells of seminiferous tubules of control rats, Bcl-2 was strongly (*p* < 0.05) expressed (Fig. 5(II.A)). But rats given AgNPs showed negative labeling of *Bcl-2* following 30 days of experiment except for the endothelial cells of tiny capillaries in the interstitial (Fig. 5(II.B)). In the testicular tissues of control rats, however, there was no cytoplasmic immunolabeling for *Caspase-3* (Fig. 5(II.C)). However, in rats given AgNPs for 30 days, Caspase-3 immunoreactivity was significantly expressed (*p* < 0.05) in the cytoplasm of all tubular lining cells (Fig. 5(II.D)).

### Molecular gene expression evaluation

**Fig. 6:**
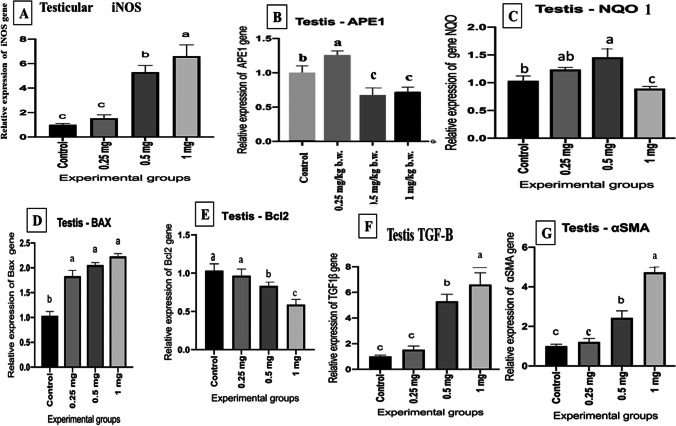
Effect of AgNPs on mRNA expression levels of (**A**) inducible nitric oxide synthase (iNOS), (**B**) apurinic/apyrimidinic endo deoxyribonuclease 1 (APE1), (**C**) NAD (P) H quinone dehydrogenase 1 (NQO1), (D) Bcl-2-associated X (BAX), (**E**) B cell lymphoma 2 (Bcl-2), (**F**) transforming growth factor (TGF-B), and alpha-smooth muscle actin (α-SMA) expression levels of the control and AgNP-treated groups after 30 days of AgNP exposure. Data are expressed as mean ± SEM (*n* = 7). Subscript letters (a, b, and c) indicate that means with different superscripts are significantly different at *p* < 0.05

The testicular *iNOS* mRNA expression levels demonstrated a significant (*p* ≤ 0.05) upregulation in the medium- and high-dose AgNP-treated groups after 30 days compared with the control group. The highest levels were found in the high-dose group (Fig. 6(A)). Moreover, testicular *APE1* expression revealed a significant increase (*p* ≤ 0.05) in rats given 0.25 mg AgNPs, while the rats received 0.5 and 1 mg AgNPs demonstrated significant downregulation of the gene compared to the control group (Fig. 6(B)). Data concerning the testicular *NQO1* expression levels showed a significant (*p* ≤ 0.05) increase in its level expression in the low- and medium-dose groups after 30 days compared with the control group. On the other hand, there was a significant downregulation in its expression level in AgNP high-dose group after 30 days in comparison with the control group (Fig. 6(C)). Moreover, the mRNA levels of the BAX gene were significantly elevated (*p* ≤ 0.05) in all AgNP-treated groups with a dose relationship as compared with the control group (Fig. 6(D)). Concerning the *Bcl-2* gene expression, when compared to the control group, *Bcl-2* gene expression levels were considerably downregulated (*p* ≤ 0.05) in medium and high AgNP-treated groups with a dosage relationship (Fig. 6(E)). Regarding the transforming growth factor (*TGF-β*), its mRNA levels were significantly upregulated (*p* ≤ 0.05) in medium and high AgNP-treated groups when compared with the control rats as demonstrated in Fig. 6(F). Moreover, the mRNA levels of the α-SMA gene showed a significant increment (*p* ≤ 0.05) in the medium and high AgNP-treated rats for 30 days as compared with the control group as illustrated in Fig. 6(G).

## Discussion

The reproductive organs are very sensitive to environmental stress like heavy metals, xenobiotics, microwaves, and nanomaterials, which have recently taken much attention (Wang et al. 2016) and the effects of NPS have been noted on the reproductive system. In vivo studies have shown body weight changes, as well as biochemical and pathological changes in animals treated with AgNPs by different routes on different organs including testes (Greco et al. 2015). Our findings revealed a significant decrease in body weight in a dose–response relationship after 15 and 30 days of administration as compared with the control group in line with De Jong et al. (2013) who stated that after a sub-acute (28-day) intravenous infusion of Ag-NP, rats showed severe growth retardation. The reduced body weight of rats could be related to a physiological change that affects the animal’s appetite and feed consumption, resulting in body weight loss. Moreover, it was also shown that injecting gold nanoparticles into mice for 10–14 days generated transitory reversible alterations in body weight (Zhang et al. 2010), in contrast to Lee et al. (2018) who found no significant dose-related bodyweight increases in rats during and after Ag–NP, Au–NP, or a combination of the two. This divergence could be explained by the NPs’ particle size (De Jong et al. 2013). Here, we also noticed a significant reduction in testicular weight in AgNP-treated groups as compared with the control group in a dose–response relationship. Changes in relative organs and body weight have been established in studies to be a sensitive indicator of the detrimental effects of drugs/chemicals or toxicants (Shittu et al. 2015). The considerable drop in the relative weight of the epididymis and testes in male rats given Ag-NP for 7 days indicates that Ag-NP caused epididymis and testes atrophy. Watanabe (2005) observed reductions in the relative weights of the seminal vesicle and prostate in relation to body weight after 19 days of Ag-NP injection in rats. The current study declared that giving rats AgNPs intraperitoneally causes alteration in serum male sex hormone and enzymes via a significant decrease in testosterone and estradiol concentrations and a significant elevation in serum enzyme activities of ALP and ACP in a dose- and time-dependent manner in all treated groups after 15 and 30 days of exposure. The higher serum ACP activities in the AgNP-treated group is a marker of benign prostatic hyperplasia and prostate cancer in its early stages, as AgNPs inhibit cholesterol transport into the inner mitochondrial membrane effectively by reducing steroidogenic acute regulatory protein (STAR) expression, stopping cholesterol conversion to pregnenolone levels (Baki et al. 2014). Our findings are in harmony with Olugbodi et al. (2020) who reported a significant decrease in LH, FSH, and testosterone levels in rats dosed with AgNPs. Also, this reduction may be due to the production of oxidative stress by NPs in the testicular tissue consequently producing adverse effects on Leydig cells which are responsible for T hormone release (Ahmed et al. 2017). Exposure to AgNPs reduced sperm motility, velocity, kinematic parameters, and LH, follicle-stimulating hormone, and testosterone concentrations in a male rat. This study suggests that AgNPs caused hormonal imbalance and oxidative stress in the testis and epididymis, thereby impacting sperm parameters (Olugbodi et al. 2020).

Semen analysis studies are important for the estimation of male reproductive performance after administration of any therapeutic agents (Graves et al. 2005) and are highly correlated with fertility (Fathi et al. 2019). Nanoparticles have different effects on sperm cell functions either upon direct exposure under in vitro conditions or if administered in vivo (Shittu et al. 2018) as the testis and epididymis are the main targets of NPs (Zhao et al. 2018). The current study showed a significant decrease in the sperm count, normal morphology, viability, and motility with an increase in the number of dead sperms in all treated after 15 and 30 days of exposure in a dose- and time-dependent manner with an increase in the number of immotile and dead sperms. The observed data are inconsistent with those of Baki et al. (2014), Wang et al. (2018), and Olugbodi et al. (2020). According to the research of Moradi-Sardareh et al. (2018), AgNPs have the potential to induce toxicity in different tissues and lead to significant changes in sperm quality and quantity.

The results of the existing study revealed exhaustion of antioxidant defense mechanism (GSH), accumulation of ROS, and lipid peroxides (MDA and NO) which in turn induces oxidative stress in the testicular tissue decreasing the vitality and potency of testes. This alteration was significantly observed in medium- and high-dose AgNP-treated groups after 30 days of exposure with a maximum significance in rats that received 1 mg AgNPs. The well-documented cellular action of Ag-NP is the liberation of free radicals and the induction of oxidative stress (Salma et al. 2011). The increased levels of H_2_O_2_ and MDA, as well as the inhibition of antioxidant enzyme activities, especially catalase, SOD, and GSH, observed in the testes and epididymis of rats dosed with Ag-NP for 7–28 days, indicate that Ag-NP penetrates the cellular organs, particularly the mitochondria, impairing the membrane potential and inducing the production of free radicals. The lowered levels of catalase, SOD, and GSH after exposure to AgNPs could be due to silver nanoparticles complexing with thiol groups (Sangodele et al. 2017) or the increased utilization of GSH, catalase, and SOD to mitigate the effects of free radicals after nanoparticle exposure (Lawal et al. 2015). Our results are consistent with Olugbodi et al. (2020) suggesting that ROS induction and oxidative damage have been involved as a reason of AgNPs toxicity (Kim et al. 2014).

Testicular histology and sperm parameters are closely linked where the alterations in the testicular structure are usually associated with changes in testicular function (Parker, 2006). Testicular histopathological changes are known as the most sensitive endpoint for detecting testicular toxicity (Lanning et al. 2002). Also, AgNPs could cross the blood-testis barrier and gather in the testes (van der Zande et al. 2012) and exert their action. The current work revealed a dose-dependent response of testicular damage, where the testicular tissues of the rats after 15 days of treatment at a dose of 0.25 mg AgNPs/kg b.w showed mild congestion, interstitial edema with mild degenerative changes of germ cells, and their sloughing into the lumen (Gozde et al. 2012). By doses 0.5 and 1 mg AgNPs/kg b.w., the lesions were degenerative; vacuolation (Sabbah et al. 2016), pyknosis, and sloughing with hypospermatogenesis rather than hyperemic with increasing the number of degenerated tubules lined with few to no spermatogenic cells. Kim et al. (1999) suggested that the number of sperms may be considered a good indicator of testicular and spermatogenic damages. The noticed multi-nucleated giant cells in the lumen of seminiferous tubules result from clumped spermatogenic cells, which lost their contact with Sertoli cells Holstein and Eckmann (1986). However, after 30 days of AgNP exposure, the lesions became more pronounced to be more degenerative and necrotic changes in a wide number of seminiferous tubules with thickening of their basement membranes (Tohamy et al. 2022). Moreover, by administration of 1 mg AgNPs, the prevalence and frequency of affected seminiferous tubules were greatly increased with remarkable necrosis. Shrunken and disordered seminiferous tubules in AgNP-treated rats were interpreted by some authors as cytotoxicity of AgNPs being linked to increased generation of ROS, which could cause apoptosis (Zhang et al. 2015). Furthermore, myoid cell contraction or deformed seminiferous tubules could cause abnormalities in the basal lamina (Mohamed et al. 2014). The observed reduction in spermatogenesis due to AgNP exposure may be attributed to the production of high concentrations of ROS, inducing oxidative damage to testicular cellular membranes; marked decrease in the number of germinal epithelial cells might cause a decrease in the number of spermatocytes and spermatids (Mohamed, 2016) or due to the obvious disruption of the interaction of the Sertoli cell (Ahmed et al. 2017). Other studies mentioned that the effect of nanoparticles on the cell cycle or release of spermatozoa to the mid duct of seminiferous tubules led to a significant decrease in sperm stem cells (Miresmaeili et al. 2013). The probable mechanism underlying AgNP toxicity in the testis has been linked to apoptosis, which has been shown to impact testicular functioning (Zhang et al. 2013). Apoptosis is a gene-regulated phenomenon regulated by death receptors, activation of caspases, mitochondrial responses, and the regulation of Bax gene expression (Ahmadian et al. 2018). AgNPs were known to induce pyknosis of nuclei of germ cells through breakdown and damage of DNA (Fathi et al. 2019). The inflammation elicited by NPS leads to ROS production and subsequently DNA damage and apoptosis (Khanna et al. 2015). Bcl-2 gene, as an apoptotic suppressor gene, while Bax is an apoptotic gene, promotes apoptosis through heterodimerization of homodimerization with Bcl-2 (Jiang et al. 2018) and the relative level of the dimerization pattern of these proteins moves the cell to survival or death (Ahmadian et al. 2018). Nano silver was demonstrated to decrease the expression of Bcl-2 and increase the expression of Bax genes in rat hippocampus, approving the involvement of the apoptosis cascade in the AgNP cytotoxicity (Shen et al. 2019). AgNPs can increase oxidative stress and DNA damage, which leads to the upregulation of p53, and its prolonged activation results in the induction of apoptosis via increasing the Bax/Bcl-2 ratio (Gholami et al. 2020). AgNPs caused a significant decrease in Bcl-2 immunostaining in the cells of seminiferous tubules of rats given 1 mg AgNPs for 30 days, which is consistent with previous research of (El-Mesalmy et al. 2021). Caspase-3 is a major effector caspase implicated in the apoptotic cascade within cells, cleaving a variety of cellular substrates and causing apoptosis (Bantel et al. 2001). In the exiting work, in comparison to control rats, *caspase-3* was immuno-expressed heavily in the testicular tissue of AgNP-injected rats. AgNPs caused apoptosis by increasing the Caspase-3/Bcl-2 ratio, which was mediated by an increase in oxidative indicators. There have been no earlier reports of the *Caspase-3/Bcl-2* pathway being involved in the harmful effects of silver nanoparticles on rats’ testes. AgNPs altered testis morphology, sperm formation, and apoptosis-related genes and proteins, including *caspase-3*, implying that this process was linked to apoptosis (Wang et al. 2021).

The current findings on the mRNA level of *Bax* showed a significant upregulation in all AgNP-treated groups with a dosage-dependent connection when compared to the control group. The *Bcl-2* gene, on the other hand, was shown to be considerably downregulated in all the treated groups, which was consistent with previous research (Tohamy et al. 2022). These findings support the idea that the *Bcl-2/Bax* pathway plays a role in AgNP cytotoxicity as seen in humans treated with AgNPs (Piao et al. 2011). Inducible nitric oxide synthase (iNOS) is a calcium-independent inducible enzyme that is activated in both normal and pathological situations (Teshfam et al. 2006) and in inflamed tissues*.* Upregulation of *iNOS* is accompanied by excessive production of NO over a prolonged period (Lirk et al. 2002). Thus, NO could induce germ cell apoptosis through the distress of the *Bax/Bcl2* regulators in the mitochondria, mediating cellular death (Vera et al, 2006). In the existing study, upregulation of the *iNOS* gene in all AgNP-treated groups after 30 days compared with control rats was noticed. *α-SMA* is a marker of myofibroblasts and has an important role in fibrosis (Tomasek et al. 2002) and is used as a marker for the fibrogenic activity of activated fibrogenic cells (Bochaton-Piallat et al. 2016). In the current work, AgNPs induced a significant upregulation of α-SMA in rats that received AgNPs in a dose-dependent manner and histological analysis revealed features of testicular fibrosis. Our result agrees with Zhou et al. (2021). TGF-B is increased and activated in fibrotic tissues in animal models, and they have been linked to the pathophysiology of fibrogenic responses in a variety of organs. TGF overexpression causes fibrotic alterations in a variety of tissues (Sonnylal et al. 2007). The mRNA level of TGF-β was dramatically elevated in the current study, boosting the fibrosis process. This suggests the linkage of TGF-β/ α-SMA in the process of fibrosis which was observed in rats administered with AgNPs for 30 days. Under normal physiological conditions, the DNA of each mammalian cell is damaged several times daily (Barnes and Lindahl, 2004). Six pathways of DNA repair have been reviewed by Damia and D’Incalci (2007); the most interesting is the Base Excision Repair (BER). Many enzymes are involved in the BER pathway. APE1 is an abundant, multifunctional, and relatively stable mammalian BER enzyme that plays a crucial role in the regulation of the cellular response to oxidative stress (Tell et al. 2009), and regulates cellular proliferative rates and keeps the stability of the genome (Vascotto et al. 2009). Impaired APEX1 activity results in unrepaired AP sites that lead to DNA strand breaks, apoptosis, and an increase in cytotoxicity (Loeb and Preston, 1986) and protect the cell from death produced by the cytotoxic and mutagenic AP sites (Fishel and Kelley, 2007). Herein, after 30 days of exposure to 0.25 mg AgNPs, a substantial upregulation of APE1 was identified in rats, showing that APE1 plays a repair role against the cellular injury induced by AgNPs. But its downregulation was detected in rats given 0.5 and 1 mg AgNPs when compared to the control non-treated group, showing that APE1 has been exhausted and its repair activity has been lost as a result of increasing the dose of AgNPs administered and the degree of the resulting toxic damage. Based on our findings and previous reports, AgNPs could induce continuous ROS generation (Salim et al. 2019) in different organelles, and more damage to nuclear and mitochondrial DNA. Our result agrees with Franchi et al. (2020) as APE1’s downregulation is correlated to an increase in DNA fragmentation and cell death rate. APE1 modulates the expression of genes involved in oxidative stress, cell proliferation, inflammation, immune response, angiogenesis, and cell death pathways through redox control of transcription factors (Shah et al. 2017). Therefore, *APE1* dysregulation in the current work could be correlated to the increased cytotoxicity of the affected cells induced by AgNPs. On the other hand, Ataya et al. (2012) noticed that *APEX1* is highly expressed in the testis of a camel as a model for mammals living in the desert to elucidate the mechanism of adaptation against high ionic radiation, temperature, and dryness. As the AgNPs enter the body, crossing the blood-testis barrier and localized in the testes, they produce inflammation and subsequent oxidative stress and production of ROS, resulting in activation of *APEX1* functions and subsequent increase in its expression as seen in rats received 0.25 mg AgNPs, to counteract the effect of AgNPs (Franchi et al. 2020) where *APEX1* and other DNA repair machinery is essential to fix mistakes and oxidized bases in DNA of the highly dividing cells, like in testis. *NQO1* is a cytosolic homomeric flavoprotein that catalyzes two-electron depletion and detoxification of quinones and their derivatives, protecting cells from oxidative stress, redox cycling, and neoplastic lesion (Dinkova-Kostova and Talalay 2000). The *NQO1* is expressed in many tissues, and regulated by the antioxidant response element (ARE) in both basal and oxidative stress conditions (Nioi and Hayes, 2004) which is regulated by the nuclear factor (erythroid-derived 2)-like 2 (*Nrf2*) via interaction with the ARE that encode *NQO1* (Bellezza et al. 2018) involved in the removal of ROS and participate to restore the redox balance. Many studies have reported that damage caused by oxidative stress can also decrease endogenous non-enzymatic antioxidants and inhibit antioxidant enzymes (Yuan et al. 2020). Increased expression of *NQO1* in response to oxidative stress provides the cell with protection strategies, reduces reactive quinones and quinone-imines to their less reactive and less toxic hydroquinones forms, and therefore blocks the formation of ROS derived from the interaction of the semiquinone with molecular oxygen (Dinkova-Kostova and Talalay 2000) or scavenge superoxide directly. In the current study, rats given 0.25 and 0.5 mg of AgNPs showed a considerable up-regulation of the *NQO1* gene after 30 days, but rats given 1 mg showed a downregulation, which is consistent with previous findings (Yuan et al. 2020; Yu et al. 2020). The observed upregulation of the *NQO1* gene could be related to the body’s defense mechanism, which involves the antioxidant system interacting with the ARE and causing antioxidant proteins to be expressed (Anwar-Mohamed et al. 2014), including *NQO1*. Furthermore, AgNPs change the expression of genes encoding antioxidant enzymes, impairing *Nrf2* signaling because of continual antioxidant protein consumption due to systemic overload caused by continuous OS synthesis (Yu et al. 2020).

## Conclusion

Long-term exposure to AgNPs at varying concentrations is thought to be extremely harmful to reproductive function and may alter animal fertility through hormonal imbalances, altered sperm parameters, oxidative stress, dysregulation of mRNA expression of oxidative stress, and apoptosis-related genes and testicular morphological changes. Furthermore, dysregulation of *NQO1* caused oxidative stress and a redox imbalance in the testes, reducing the ability to counteract AgNP-induced oxidative stress and enhancing AgNP-induced cell apoptosis via the *Bcl-2/Caspase 3* pathway, ultimately leading to apoptosis in spermatogonia and spermatocytes and resulting in alterations in testis histological structure and sperm production. Also, AgNP administration diminished the physiological repair mechanism through downregulation of *APE1* mRNA and triggering the process of fibrosis via *TGF-β/α-SMA* upregulation.

## Supplementary Information

Below is the link to the electronic supplementary material.

Supplementary file1 (DOCX 562 KB)

## Data Availability

The authors confirm that the data supporting the findings of this study are available within the article and/or its supplementary materials.

## Abbreviations

AgNPs: Silver nanoparticles
MDA: Malondialdehyde
NO: Nitric oxide
ROS: Reactive oxygen species
GSH: Reduced glutathione
ACP: Acid phosphatase
ALP: Alkaline phosphatase
