# Supplementary figures and images for: Silver nanoparticles induced testicular damage targeting NQO1 and APE1 dysregulation, apoptosis via Bax/Bcl-2 pathway, fibrosis via TGF-β/α-SMA upregulation in rats
Source: Environ Sci Pollut Res Int. 2022 Nov 11;30(10):26308–26. doi: 10.1007/s11356-022-23876-y (PMC9995601; doi:10.1007/s11356-022-23876-y)

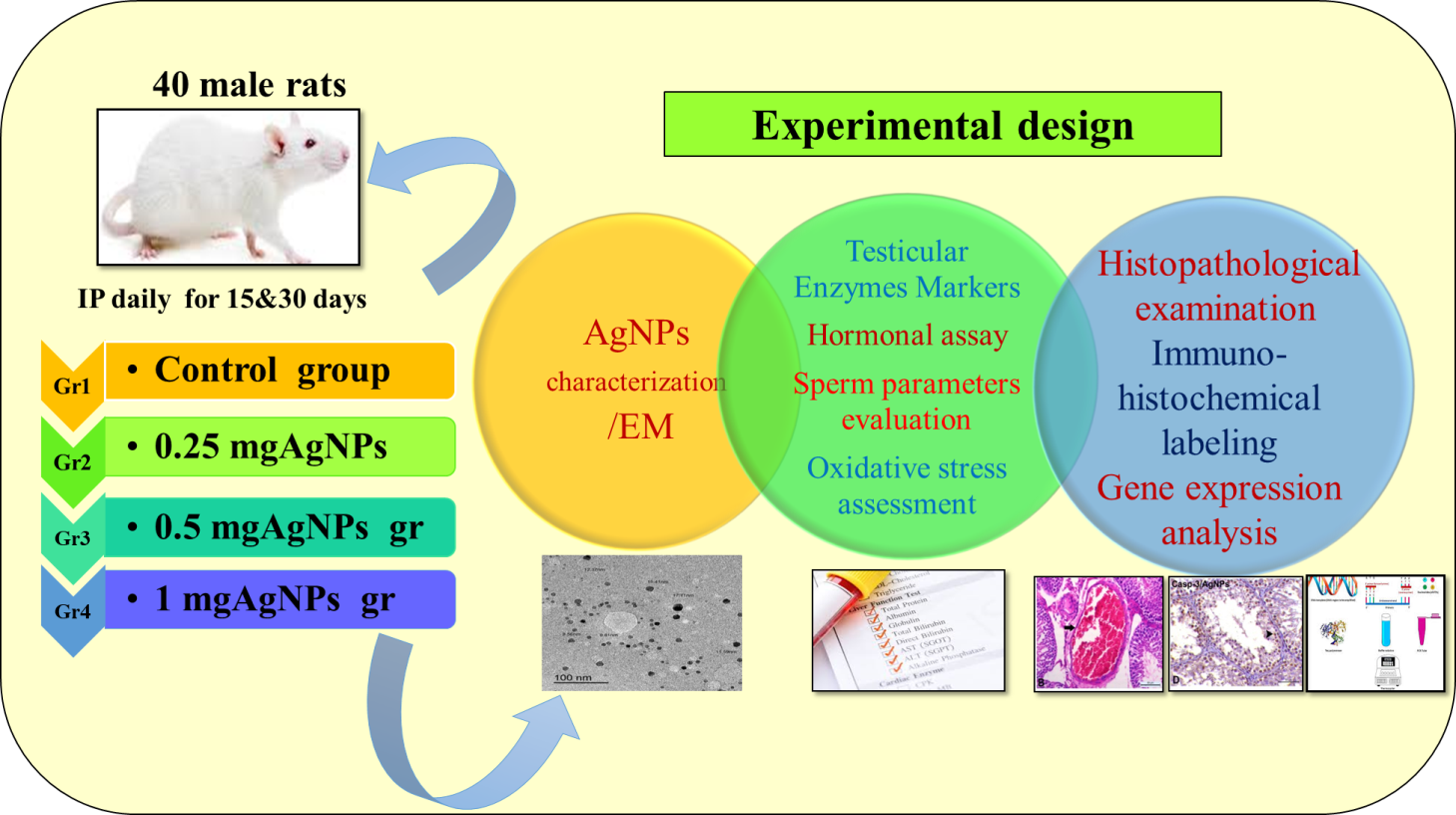


Figure.S1: Experimental design

Supplement: Supplementary file 1 — Supplementary file1 (DOCX 562 KB) [file 11356_2022_23876_MOESM1_ESM.docx]
